# Pittsburgh Conference, New Orleans, USA, 1988

**DOI:** 10.1155/S146392468800029X

**Published:** 1988


Journal of Automatic Chemistry, Vol. 10, No. 3 (July–September 1988), pp. 158–161

### Pittsburgh Conference, New Orleans, USA, 1988

*Some of the products launched at the 39th Pittsburgh Conference and Exposition (22–25 February 1988) are detailed below. The exposition this year involved 830 companies using 2300 booths and 39 seminar rooms.*

### Pittcon launch: Automated tumble mixing station

The Zymark Corporation introduced a new automated tumble mixing station for use with the Zymate II Laboratory Robot at the 1988 Pittsburgh Conference. The tumble mixing station has been designed to work with other Zymate Laboratory Stations to automate Liquid/Liquid (L/L) extractions. This new station can be used to prepare samples for subsequent HPLC or GC analysis, or any other method that requires L/L extractions in the range of 1–15 ml. The tumble mixing station has an integral multi-tube cap sealing mechanism which permits samples to be extracted without performing time-consuming screw-capping operations.

Typical applications for this station include:

- Pharmaceutical animal toxicology studies.
- Clinical drug metabolism studies.
- Pesticide residue sample preparation.
- General trace component GC or HPLC sample preparation.

*Details from Inquires Manager, Zymark Corporation, Zymark Center, Hopkinton, Massachusetts 01748, USA.*

### Pittcon launch: HP 1050

A new series of HPLC modules was launched by Hewlett-Packard at the 1988 Pittsburgh Conference and Exposition held in New Orleans. The HP 1050 series modules may be incorporated into existing modular HPLC systems, and are designed to new standards of reliability – Hewlett-Packard offers an optional 99% guaranteed uptime service for each of the HP 1050 series products.

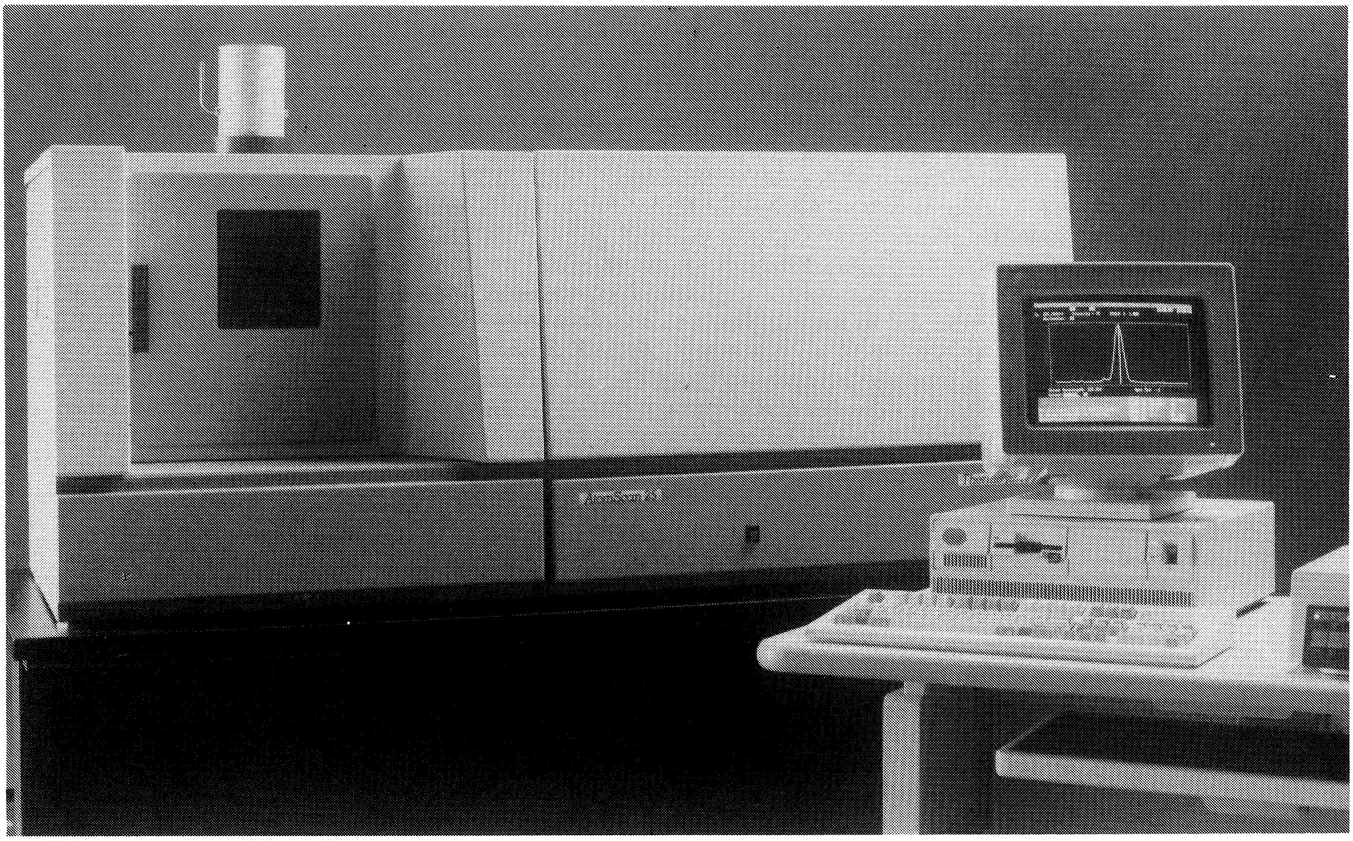

*The AtomScan 25 Spectrometer which was shown for the first time at the 39th Pittsburgh Conference in New Orleans on 22–25 February 1988. Its manufacturer, Thermo Jarrell Ash, produces atomic absorption, plasma emission, and arc/spark emission spectrometers used for elemental analysis of water, oil, metal, geological, biological, and other materials.*

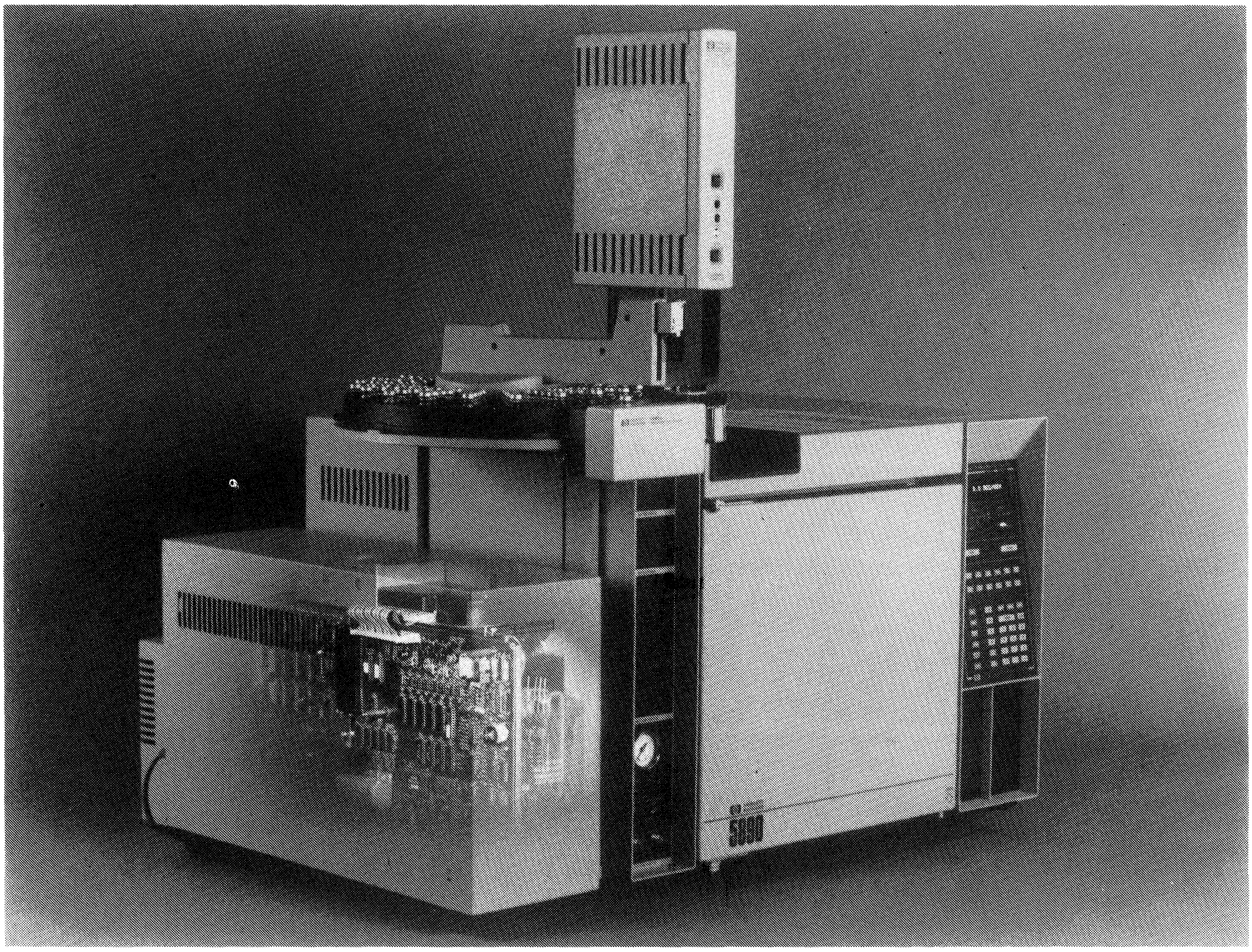

*Believed to be the smallest, simplest mass spectrometer on the market today, the HP 5971A mass selective detector (MSD) was launched by Hewlett-Packard at the 1988 Pittsburgh Conference and Exposition. Designed for GC/MS analyses, the MSD mounts on the side of the HP 5890A gas chromatograph, providing true, classical electron impact (EI) spectra while taking up 7 in of bench space. Total ion scanning and selective ion monitoring may be carried out, and the easily-maintained system is optimized for capillary chromatography. Details from Tina Mears, Analytical Instrument Group, Hewlett-Packard Ltd, Miller House, The Ring, Bracknell, Berkshire. RG12 1XN, UK. Tel.: 0344 424898.*

The five current modules in the HP 1050 series are designed with standardized user-interface and module-to-module communication, so that each may be incorporated into most modular HPLC systems. The HP 1050 modules include two pumping systems, two detectors and an autosampler. The isocratic pump uses a serial dual-piston method of delivering solvent, with variable stroke volume to ensure flow stability across a broad flow range. This pump is used as the basis of the quaternary pumping system, which incorporates a high-speed proportioning valve to mix the mobile phase at low pressure. This provides gradient capability for up to four solvents. A separate solvent cabinet degasses and filters the solvents.

A high sensitivity, programmable variable wavelength detector features wavelength switching and stop-flow scanning. Interchangeable cells allow this module to be used for a wide range of different HPLC applications. The multiple wavelength detector uses diode array technology for dual-wavelength monitoring and scanning without stopping the flow. Chromatographic data and peak purity information may be obtained simultaneously.

The autosampler features a precision injection mechanism that can handle volumes of up to 400 μl. Injection volumes are programmable, and calibration routines are independent of sample position and variable sample capacity. A 21-sample tray is standard; a 100-sample tray is optional.

Each module is designed with the minimum number of mechanical parts, so that failure rates should be low. They can be simply installed, operated and serviced by laboratory personnel. Electronic diagnostic signal routine maintenance, and access to parts requiring maintenance is easy. Running costs are low: a service contract costs 3–4% of the module list price.

*Enquiries to Tina Mears, Analytical Instrument Group, Hewlett-Packard Ltd, Miller House, The Ring, Bracknell, Berkshire RG12 1XN, UK. Tel.: 0344 424898.*

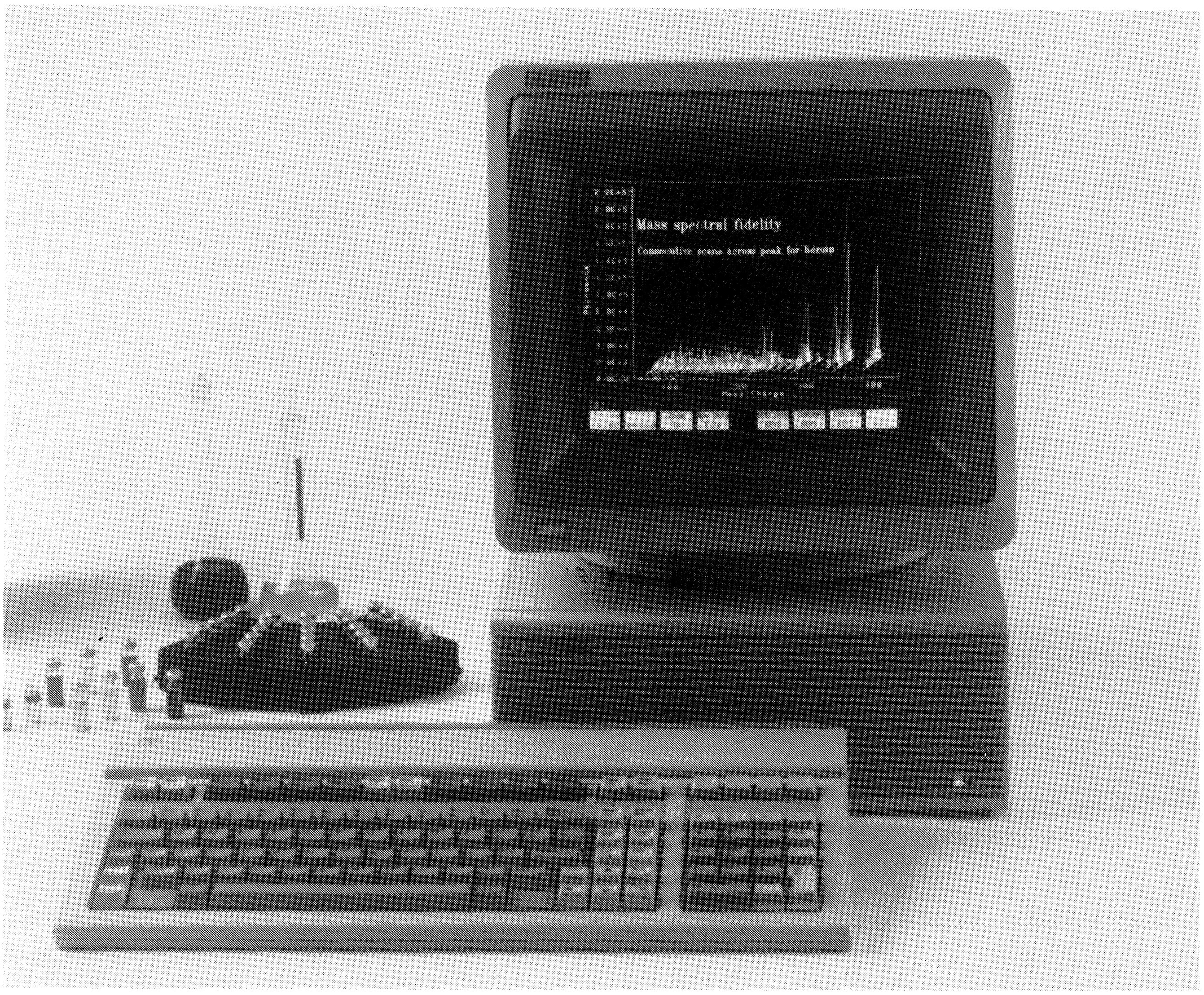

*Together with the UNIX-based MS ChemStation launched last month at Pittcon, Hewlett-Packard also introduced new software for its Pascal series HP 59770C MS ChemStation. The new software Revision 3·2 provides enhanced graphics, mathematics, statistics, automation and library search capabilities. The software includes a complete set of applications for mass selective detectors, GC/MS and LC/MS systems, including tuning, data acquisition, data editing and reporting. Details from Tina Mears, Hewlett-Packard Ltd* *(see p. 159).*

### Sequential ICP

The AtomScan 25 Spectrometer (introduced at Pittcon 88) is a sequential scanning plasma emission spectrometer for elemental analysis of water, oil, and other materials. The spectrometer features an extremely fast and reliable galvanometer drive scanning mechanism, which provides both high resolution and wide wavelength coverage. It is operated from an IBM System 2 computer running ThermoSPEC software, which presents a consistent windowed operator interface with function key operation and help screens. The spectrometer is totally automated, with computer control over the sample introduction, plasma discharge, optical, and read-out systems. Unattended operation from an autosampler provides all the quality control logic functions required by the EPA Contract Laboratory Program. The software also includes Multiquant, a fast semi-quantitative analysis program, and Enable, an integrated word-processor, data-base manager, spreadsheet, graphics, and telecommunications package.

*Additional details from Thermo Jarrell Ash Corporation, 8E Forge Parkway, Franklin, Massachusetts 02038, USA. Tel.: 617 520 1880; Fax: 617 520 1758.*

### Analysis of drugs in biological fluids

The Zymark Corporation also introduced a new automated system for the analysis of drugs in biological fluids at Pittcon. This new system was designed for laboratories that perform testing of biological fluids (primarily blood plasma or urine) for drugs and metabolites in preclinical studies and clinical trials of new drugs.

The new system is based on the Zymate II Laboratory Robotics System and provides automated preparation and injection of samples for HPLC or GC analysis.

*Multiple method capability*

Automated techniques for both Liquid/Liquid (L/L) and Liquid/Solid (L/S) Extraction are provided in this system. These techniques may be used separately or combined in the same procedure. This system has been designed to provide convenient multiple method operation. For example toxicology samples from a preclinical study that are being prepared for HPLC analysis using L/L Extraction may be prepared on the same system that is also being used to prepare drug metabolism samples from clinical trials of another drug using L/S Extraction.

The automated, programmable features of the system include:

- Cooled storage of incoming samples.
- Accurate gravimetric-based sample transfers.
- Addition of internal standard.
- Addition of buffer.
- Liquid/Liquid extraction.
- Liquid/Solid extraction.
- Evaporation.
- Reconstitution.
- Filtration.
- LC injection or finished sample storage in vials.

*Improved laboratory productivity*

This new system permits more samples to be processed at the same staffing levels and a reduction of human sample preparation errors minimizes the amount of retesting that is required. Together, these factors provide a significant productivity gain for laboratories performing toxicology, bioavailability, drug metabolism, or pharmacokinetic studies.

*Validation and documentation*

Even more importantly, this system provides a machine-generated audit trail of exactly how each sample was processed including any critical timing factors. This audit trail speeds the chemistry validation process and provides excellent documentation for the subsequent review and approval of data.

*US list price and delivery*

The US list price for a Zymate II System configured for automated drug metabolism is in the range of $60 000–$75 000 depending on the specific system configuration. This product is available through Zymarks's US and international sales network.

*Details from Zymate (see p. 158).*

### THE 40th PITTSBURGH CONFERENCE AND EXPOSITION ON ANALYTICAL CHEMISTRY AND APPLIED SPECTROSCOPY

*To be held from 6 to 10 March 1989 in the Georgia World Congress Center, Atlanta, USA*

The 1988 Pittsburgh Conference technical programme contained extensive coverage of new and sophisticated developments, for example in scanning tunneling microscopy, capillary zone electrophoresis and modified electrodes. Over 25 000 conferees attended 34 symposia, including three general interest symposia and two UMIX symposia. The 1134 contributed presentations were supplemented by 149 posters.

The 1989 program will continue to offer technical sessions in all areas of science related to analytical chemistry and spectroscopy. Of particular interest in 1989 will be symposia covering the contributions of analytical chemistry and spectroscopy to the life sciences and materials research.

Abstracts for the 1989 Pittsburgh Conference technical programme have now been invited. Deadlines and rules for submission are as follows:

**3 August 1988** is the deadline for receipt of abstracts.

One copy of a 250-word abstract must be submitted for review. All abstracts will be carefully evaluated. The abstract should clearly state (*a*) the objective of the work, (*b*) equipment and procedures used, and (*c*) results and conclusions. Abstracts must include sufficient content for adequate evaluation by the Conference Program Committee.

In November 1988 the designated speaker will be informed of the paper's status: accepted, on the waiting list, or rejected.

Specific references to vendor products in the titles of papers are not permitted and will be automatically deleted.

A second abstract will be required for reproduction in book form for distribution to the conferees. Forms and instructions concerning this second abstract will be sent to the designated speaker with the notification of acceptance of the paper. The second abstract will be due on **14 December 1988.** The title of the paper and the author information originally submitted cannot be changed for the second abstract.

*More information from Alma Johnson, Pittcon Program Secretary, 12 Federal Drive, Suite 322, Pittsburgh, Pennsylvania 15235, USA.*

